# Gastroprotective Effect of Juanislamin on Ethanol-Induced Gastric Lesions in Rats: Role of Prostaglandins, Nitric Oxide and Sulfhydryl Groups in the Mechanism of Action

**DOI:** 10.3390/molecules25092246

**Published:** 2020-05-10

**Authors:** María Elena Sánchez-Mendoza, Yaraset López-Lorenzo, Leticia Cruz-Antonio, Arturo Cruz-Oseguera, Jazmín García-Machorro, Jesús Arrieta

**Affiliations:** 1 Escuela Superior de Medicina, Instituto Politécnico Nacional, Plan de San Luis y Díaz Mirón, Colonia Casco de Santo Tomás, Miguel Hidalgo, Ciudad de México 11340, Mexico; mesmendoza@hotmail.com (M.E.S.-M.); yarlop_2310@outlook.com (Y.L.-L.); cuoa_@hotmail.com (A.C.-O.); jazzgama@hotmail.com (J.G.-M.); 2 Facultad de Estudios Superiores Zaragoza, UNAM. Av. Guelatao No. 66, Colonia Ejército de Oriente, Iztapalapa, Ciudad de México 09230, Ciudad de Mexico; letycruza@yahoo.com.mx

**Keywords:** *Calea urticifolia*, juanislamin, sesquiterpene lactone

## Abstract

Peptic ulcer disease, the most common gastrointestinal disorder, is currently treated with several types of drugs, but all have severe side effects. The aim of the present study was to evaluate the gastroprotective activity of juanislamin, isolated from *Calea urticifolia*, in a rat model of ethanol-induced gastric lesions. Thirty minutes after orally administering a given dose of juanislamin (from 1 to 30 mg/kg) or carbenoxolone (the reference drug, at 1–100 mg/kg) to rats, 1 mL of ethanol was applied, and the animals were sacrificed 2 h later. The stomachs were removed and opened to measure the total area of lesions in each. To examine the possible participation of prostaglandins, nitric oxide and/or sulfhydryl groups in the mechanism of action of juanislamin, the rats received indomethacin, NG-Nitro-l-arginine methyl ester hydrochloride (l-NAME) or *N*-ethylmaleimide pretreatment, respectively, before being given juanislamin and undergoing the rest of the methodology. Juanislamin inhibited gastric lesions produced by ethanol in a non-dose-dependent manner, showing the maximum gastroprotective effect (100%) at 10 mg/kg. The activity of juanislamin was not modified by pretreatment with indomethacin, l-NAME or *N*-ethylmaleimide. In conclusion, juanislamin protected the gastric mucosa from ethanol-induced damage, and its mechanism of action apparently does not involve prostaglandins, nitric oxide or sulfhydryl groups.

## 1. Introduction

Peptic ulcers are sores or lesions in the gastrointestinal mucosa extending throughout the muscularis mucosae, typically characterized by different stages of necrosis and affected by neutrophil infiltration, reduced blood flow, and increased oxidative stress and inflammation. Usually, peptic ulcers are found in the stomach and proximal duodenum, and less frequently in the lower third of the esophagus, distal duodenum and jejunum [1]. The pathophysiology of peptic ulcers can be considered as an imbalance between aggressive factors (e.g., hydrochloric acid, pepsin and *Helicobacter pylori* infection) and local defense mechanisms of the gastric mucosa, including proper blood flow and secretion of mucus, bicarbonate, prostaglandins, nitric oxide and sulfhydryl groups [2]. Due to the considerable rise in the incidence of peptic ulcers and their complications in recent years, they are now a major cause of morbidity and mortality worldwide [3].

The therapeutic strategies for managing peptic ulcers are based on eliminating pain, healing ulceration, preventing recurrence, and, more recently, eradicating *Helicobacter pylori*. The drugs administered to carry out these strategies are classified according to their mechanism of action, being antacids, cytoprotectants and antisecretory agents [4]. Among the latter, the most promising for treating gastric ulcers are inhibitors of the H^+^/K^+^ exchange pump. However, it has been shown that prolonged use of this type of inhibitor (e.g., omeprazole) can lead to a decreased absorption of vitamin B12, which in turn may result in dementia, neurological damage, anemia or other possibly irreversible complications. Additionally, proton pump inhibitors have been implicated in acute myocardial infarction [5] and pancreatic cancer [6]. The foregoing overview highlights the need to develop new types of gastroprotective therapies.

Medicinal plants are often the source of new drugs. From one such plant, *Calea urticifolia*, three compounds with gastroprotective activity have been isolated and previously reported [7,8,9]. The aim of the present study was to evaluate the gastroprotective effect of juanislamin (PubChem CID 6440536), isolated from *C. urticifolia*, in a rat model of ethanol-induced gastric lesions. The mechanism of action was explored by examining the possible participation of nitric oxide, prostaglandins and sulfhydryl groups.

## 2. Results

### 2.1. Ethanol-Induced Gastric Lesions

**Figure 1:**
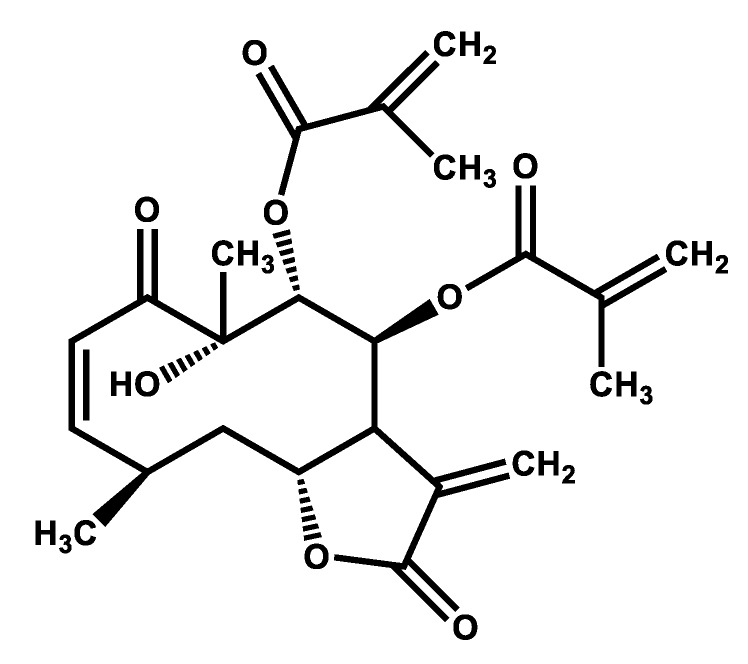
The structure of juanislamin.

**Figure 2:**
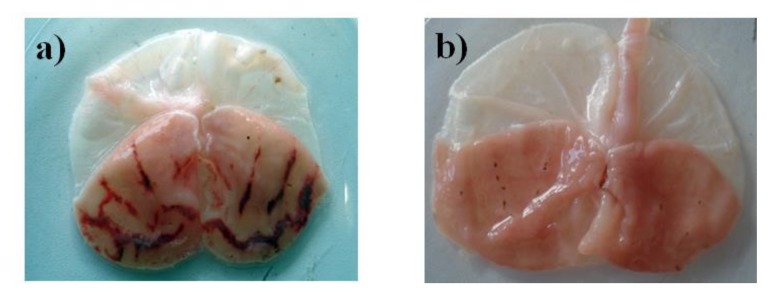
Representative images of gastric mucosal lesions in rats treated with (**a**) the vehicle plus ethanol and (**b**) juanislamin plus ethanol.

**Figure 3:**
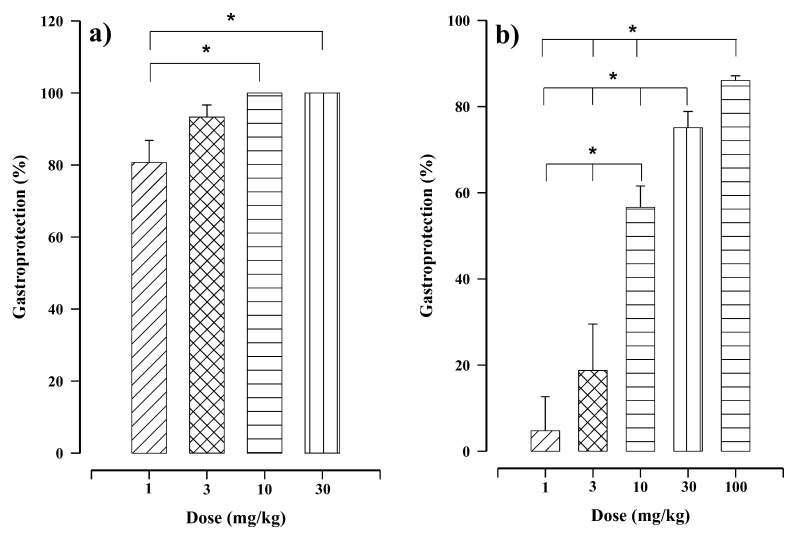
In the rat model of ethanol-induced gastric lesions, distinct levels of gastroprotection resulted from different treatments: (**a**) juanislamin (1–30 mg/kg) and (**b**) carbenoxolone (1–100 mg/kg). Bars represent the mean ± SEM (*n* = 7). * *p* ≤ 0.05, Kruskal–Wallis test followed by Dunn’s multiple comparison.

Oral administration of juanislamin (Figure 1; 1–30 mg/kg) reduced ethanol-induced gastric injury (Figure 2), inhibiting gastric lesions in a non-dose-dependent manner (Figure 3a). Maximum gastroprotection (100%) was achieved with the dose of 10 mg/kg. The gastroprotection provided by the dose of 3 mg/kg (93.32 ± 3.34%) was slightly lower but not significantly different, and that afforded at 1 mg/kg was 80.65 ± 6.16%. Carbenoxolone, on the other hand, exhibited dose-dependent activity (Figure 3b), reaching the maximum gastroprotection at 100 mg/kg (86.06 ± 1.08%). Whereas the protective effects produced by the doses of 30 and 10 mg/kg were 75.09 ± 3.76% and 56.6 ± 4.96%, respectively, those furnished by the doses of 3 and 1 mg/kg were minimal. As can be appreciated, carbenoxolone appears to be less potent than juanislamin.

### 2.2. Participation of Prostaglandins, Nitric Oxide and Sulfhydryl Groups in the Mechanism Action of Juanislamin

**Figure 4:**
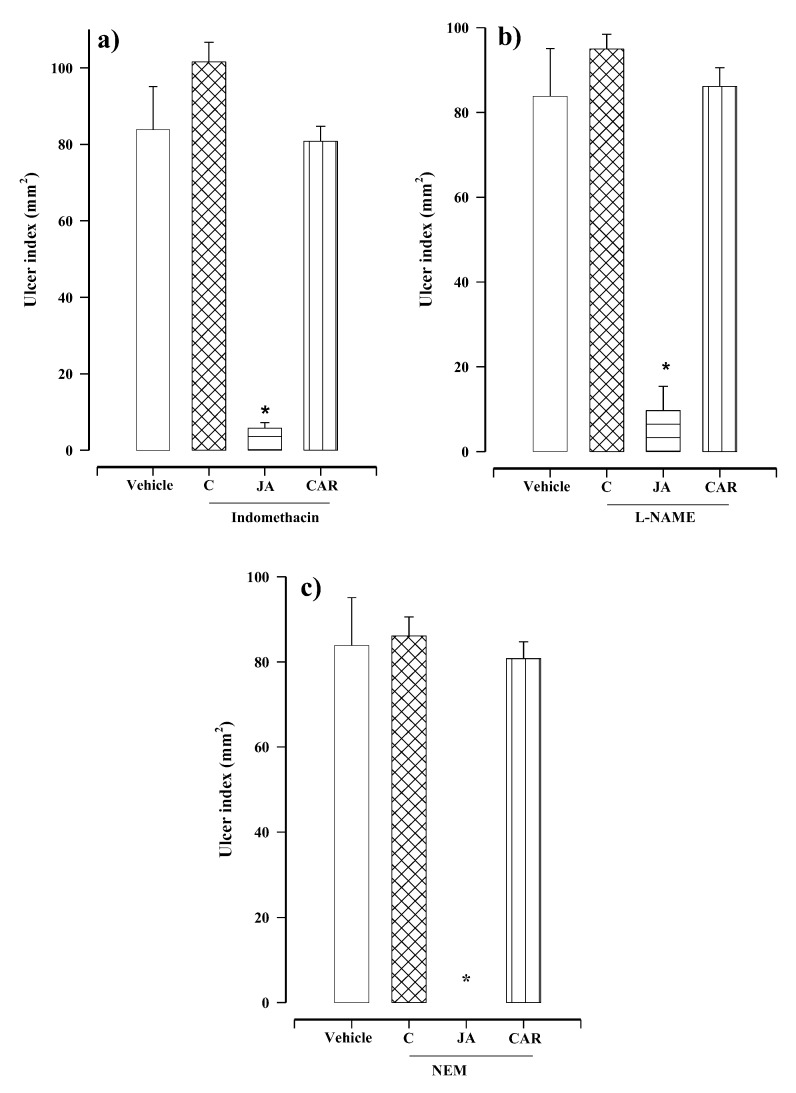
Gastroprotective effect of juanislamin (JA, at 10 mg/kg) and carbenoxolone (CAR, at 100 mg/kg) in rats receiving one of three pretreatments: (**a**) indomethacin (10 mg/kg), (**b**) NG-Nitro-l-arginine methyl ester hydrochloride (l-NAME, 70 mg/kg) or (**c**) *N*-Ethylmaleimide (NEM, 10 mg/kg). Comparisons were made to the negative control (vehicle + ethanol). C = different inhibitors plus ethanol. Bars represent the mean ± SEM (*n* = 7). * *p* ≤ 0.05, Kruskal–Wallis test followed by Dunn’s multiple comparisons.

The mechanism of action of juanislamin was explored by pretreatments with indomethacin (a prostaglandin inhibitor), NG-Nitro-l-arginine methyl ester hydrochloride (l-NAME, a nitric oxide synthase inhibitor) and *N*-ethylmaleimide (NEM, a blocker of sulfhydryl groups) [10]. The rats were pretreated with one of the aforementioned inhibitors and then ethanol was applied (without administering the test or reference compound). The data reveal the lack of significant difference when comparing the ulcer index of the control group of rats receiving 0.05% Tween 80 plus ethanol (83.33 ± 11.26 mm^2^) to that found in animals pretreated with indomethacin (101 ± 5.16 mm^2^), l-NAME (94.96 ± 3.46 mm^2^) or NEM (86.44 ± 4.45 mm^2^) and then given ethanol (Figure 4a–c). Therefore, the gastric damage produced in the present study cannot be attributed to any of the three inhibitors at the doses herein employed. 

Independent groups of rats were pretreated with indomethacin (10 mg/kg), l-NAME (70 mg/kg) or NEM (10 mg/kg), and subsequently treated with juanislamin (10 mg/kg) plus ethanol. The resulting ulcer rates were 5.75 ± 1.47, 9.68 ± 5.71 and 0.0 ± 0.0 mm^2^, respectively. Since these values are significantly different from the 83.33 ± 11.26 mm^2^ gastric damage observed in the control group of animals (vehicle plus ethanol), prostaglandin, nitric oxide and non-protein sulfhydryl are not involved in the gastric protection of juanislamin (Figure 4a–c). Regarding carbenoxolone, pretreatment with each of the three inhibitors reversed its effect (Figure 4a–c), as evidenced by the respective ulcer indexes (79.76 ± 3.95, 86.11 ± 4.45 and 80.76 ± 3.95 mm^2^). These data are in agreement with reports in the literature [11]. 

## 3. Discussion

Gastric ulcers are characterized by lesions of the gastric mucosa caused by alterations in the balance between aggressive factors and local protection of the gastric mucosa [12]. Tobacco smoking, the use of non-steroidal anti-inflammatory drugs (NSAIDs) and the consumption of alcohol are the principal risk factors for gastric ulcers [13]. Since current treatments for this disorder lead to serious side effects, great efforts have been made to find less toxic alternatives. In general, medicinal plants are an attractive source of new drugs. A plant with known gastroprotective activity, *C. urticifolia* [7,8,9], was herein processed to isolate juanislamin, which was evaluated for gastroprotection in a model of ethanol-induced gastric lesions. Assays were carried out to explore the possible contributions of prostaglandins, nitric oxide and sulfhydryl groups in the mechanism of action of the compound under study.

Gastric damage produced by ethanol involves many factors of imbalance, including the generation of free radicals and DNA damage, a decrease in the concentration of glutathione, and alterations in the mucus/bicarbonate layer [14]. Oral administration of juanislamin at all doses herein tested provided substantial protection against ethanol-induced gastric lesions, attaining 100% gastroprotection at 10 mg/kg (Figure 3a). Our group has previously described a similar effect with 2,3-epoxyjuanislamin, calealactone B and calein D (sesquiterpene lactones with a germacrane skeleton) also isolated from *C. urticifolia* [7,8,9]. However, these three compounds all showed potencies slightly lower than that of juanislamin. In all three cases, a dose of 30 mg/kg was required to reach 100% gastroprotection. Regarding the structural differences, juanislamin contains an additional α,β-unsaturated carbonyl group compared to the other three compounds. This moiety seems to have important biological activity.

Prostaglandins protect the gastric mucosa by promoting mucus/bicarbonate secretion, maintaining blood flow and limiting acid secretion [15]. The possible participation of these compounds in gastroprotection is generally explored by using indomethacin, a prostaglandin inhibitor [7]. Since indomethacin pretreatment did not diminish the gastroprotection provided by juanislamin (Figure 4a), prostaglandins do not take part in its mechanism of action. Contrarily, indomethacin sharply reduced the gastroprotection of the reference drug, as observed in other studies [8]. 

Nitric oxide also plays a key role in the protection of the gastric mucosa by regulating blood flow in the tissue and significantly contributing to mucus/bicarbonate secretion [16]. The inhibition of nitric oxide synthesis found presently by the administration of L-NAME did not modify the gastroprotective activity of juanislamin (Figure 4b). Consequently, the mechanism of action of the test compound is not related to nitric oxide. For carbenoxolone, however, gastroprotection was notably decreased by l-NAME pretreatment, as previously reported [8].

Sulfhydryl groups protect the gastric mucosa by keeping the gastric mucus stable through the formation of disulfide bridges and the elimination of free radicals. The latter are capable of causing lipid peroxidation [17]. Following pretreatment with NEM in the current contribution, there was no significant change in the protection furnished by juanislamin against ethanol-induced gastric lesions (Figure 4c). Thus, the mechanism of action of gastroprotection does not involve sulfhydryl groups. Contrarily, the effect of carbenoxolone was reversed by NEM pretreatment, coinciding with published results. None of the three gastroprotective factors examined herein take part in the mechanism of action of juanislamin or the other three compounds isolated from *C. urticifolia* that have shown gastroprotective activity [7,8,9]. 

In one study, lactones isolated from *C. urticifolia* were found to bind to the sulfhydryl groups of the cysteine residues of the nuclear factor (erythroid-derived 2)-like 2 (Nrf2) system [18]. Interestingly, the products of omeprazole metabolism inhibit ATPase H^+^/K^+^ by binding to its cysteine residues [19]. Hence, juanislamin may also bind to cysteine residues and trigger antioxidant protective activity or inhibit ATPase such as omeprazole. Further research is needed to explore the mechanism of action of this compound.

## 4. Materials and Methods 

### 4.1. Animals 

Assays were carried out with 55- to 60-day-old male Wistar rats (180–220 g) provided by the Facultad de Estudios Superiores Zaragoza, UNAM. The animals were treated in accordance with the Official Mexican Norm for the Care and Handling of Animals (NOM-062-ZOO-1999) and international standards for the use of laboratory animals. They were housed in individual plastic containers and given free access to food and water, except during the 24 h before experiments when food was removed. All experiments involved 7 animals per group and these were randomized for each treatment. 

### 4.2. Drugs

Carbenoxolone (PubChem CID: 636403, catalog number: C4790), N^G^-Nitro-l-arginine methyl ester hydrochloride (PubChem CID: 39836, catalog number: N5751), *N*-Ethylmaleimide (PubChem CID: 4362, catalog number: E3876) and indomethacin (PubChem CID: 3715, catalog number: I7378) were purchased from Sigma Chemical (St. Louis MO., USA). The compounds were freshly elaborated prior to each administration.

### 4.3. Isolation of Juanislamin

The dichloromethane extract was prepared by macerating plant leaves (6.7 kg), as described by Sánchez-Mendoza et al. [10]. Column chromatography was performed with 300 g of the extract and an elution system based on hexane and mixtures of hexane/dichloromethane in various proportions. White crystals (1.5 g) were obtained from the fractions 80–90 (hexane/dichloromethane, 5:5) and identified as juanislamin (Figure 1) by comparing the ^1^H- and ^13^C-NMR spectra to those previously published [20]. Compound CID: 6440536, according IUPAC name: [(3aS,4S,5R,6R,8Z,10R,11aR)-6-hydroxy-6,10-dimethyl-3-methylidene-5-(2-methylprop-2-enoyloxy)-2,7-dioxo-3a,4,5,10,11,11a-hexahydrocyclodeca[b]furan-4-yl] 2-methylprop-2-enoate.

### 4.4. Ethanol-Induced Gastric Lesions

Independent groups of rats were orally administered juanislamin or carbenoxolone (the reference compound) at different doses in a volume of 0.5 mL/100 g. Juanislamin was suspended in 0.05% Tween 80 and applied at 1–30 mg/kg. Carbenoxolone was dissolved in distilled water and delivered at 1–100 mg/kg. At 30 min post-treatment, 1 mL of ethanol was orally applied to all animals, regardless of weight. The animals were sacrificed 2 h later and the stomachs were immediately dissected, filled with 2% formaldehyde, and then placed in a container with the same solution for 5 min. Subsequently, each stomach was opened along the greater curvature and the area of the lesions was blindly measured by using a stereoscopic microscope (×10) equipped with an ocular micrometer. The sum of the area of the lesions of each stomach represents the ulcer index. Gastroprotection (%) was calculated according to:

% gastroprotection = (UIC − UIT) × 100/UIC

where UIC and UIT are the ulcer indexes of the control and experimental groups, respectively [21].

### 4.5. Testing for the Participation of Prostaglandins

To investigate the possible involvement of prostaglandins in the mechanism of action of juanislamin, three groups were subcutaneously injected with indomethacin (10 mg/kg) dissolved in saline solution with 5 mM NaHCO_3_ (0.1 mL/100 g) and one control group with the saline solution only (using the same route and volume). After 75 min, the following treatments were administered orally: Tween 80 (0.5%, 0.5 mL/100 g) to the control group and one of the indomethacin groups, as well as juanislamin (10 mg/kg) or carbenoxolone (100 mg/kg) to the other two indomethacin groups. At 30 min post-treatment, all animals were given 1 mL of ethanol and 2 h later were sacrificed. The stomachs were immediately dissected and processed (as previously mentioned) to determine the ulcer index. 

### 4.6. Testing for the Participation of Nitric Oxide

To examine the role of nitric oxide in the mechanism of action of juanislamin, three groups were intraperitoneally injected with L-NAME (70 mg/kg) dissolved in saline solution (0.1 mL/100g) and one control group with saline solution. After 30 min, the control animals and one of the l-NAME groups were administered Tween 80 (0.05%). The other two l-NAME groups were treated with juanislamin (10 mg/kg) or carbenoxolone (100 mg/kg). At 30 min post-treatment, all animals were given 1 mL of ethanol and 2 h later were sacrificed. The stomachs were immediately dissected and processed (as previously mentioned) to determine the ulcer index.

### 4.7. Testing for the Participation of Sulfhydryl Groups

To explore the possible contribution of sulfhydryl groups in the mechanism of action of juanislamin, NEM was subcutaneously injected (10 mg/kg dissolved in saline, 0.1 mL/100 g) to three groups of animals and saline solution to one control group. After 30 min, the control animals and one of the NEM groups were administered Tween 80 (0.05%). The other two NEM groups were treated with juanislamin (10 mg/kg) or carbenoxolone (100 mg/kg). At 30 min post-treatment, all animals were given ethanol and 2 h later were sacrificed. The stomachs were immediately dissected and processed (as previously mentioned) to determine the ulcer index.

### 4.8. Statistical Analysis

Data are expressed as the mean ± SEM (n = 7). The statistical significance between treatments was examined by the Kruskal–Wallis test followed by Dunn’s multiple comparison test, with significance considered at *p* ≤ 0.05.

## 5. Conclusions

In summary, the results from the evaluation of the gastroprotective activity of juanislamin reinforce the idea that sesquiterpene lactones with a germacrane skeleton could possibly be instrumental in the search for new drugs to treat gastric ulcers. Apparently, the mechanism of action of this type compound does not involve prostaglandins, nitric oxide or sulfhydryl groups.

## Abbreviations

NEM	*N*-Ethylmaleimide
L-NAME	NG-Nitro-l-arginine methyl ester hydrochloride
NSAIDs	Non-steroidal anti-inflammatory drugs
SEM	Standard error of the mean
Nrf2	Nuclear factor (erythroid-derived 2)-like 2
